# Upregulation of Phosphatase 1 Nuclear-Targeting Subunit (PNUTS) Is an Independent Predictor of Poor Prognosis in Prostate Cancer

**DOI:** 10.1155/2020/7050146

**Published:** 2020-04-25

**Authors:** Andreas Marx, Andreas M. Luebke, Till S. Clauditz, Stefan Steurer, Christoph Fraune, Claudia Hube-Magg, Franziska Büscheck, Doris Höflmayer, Maria Christina Tsourlakis, Christina Möller-Koop, Ronald Simon, Guido Sauter, Cosima Göbel, Patrick Lebok, David Dum, Simon Kind, Sarah Minner, Jakob Izbicki, Thorsten Schlomm, Hartwig Huland, Hans Heinzer, Eike Burandt, Alexander Haese, Markus Graefen, Jan Meiners

**Affiliations:** ^1^Institute of Pathology, Clinical Center Fürth, Jakob-Henle-Straße 1, 90766 Fürth, Germany; ^2^Institute of Pathology, University Medical Center Hamburg-Eppendorf, 20246 Hamburg, Germany; ^3^General, Visceral and Thoracic Surgery Department and Clinic, University Medical Center Hamburg-Eppendorf, 20246 Hamburg, Germany; ^4^Department of Urology, Charité-Universitätsmedizin Berlin, 10117 Berlin, Germany; ^5^Martini-Clinic, Prostate Cancer Center, University Medical Center Hamburg-Eppendorf, 20246 Hamburg, Germany

## Abstract

Protein phosphatase 1 nuclear-targeting subunit (PNUTS) is ubiquitously expressed and associates with PTEN and protein phosphatase 1 (PP1) to control its activity. The role of PNUTS overexpression has hardly been studied in cancer. In this study, we used immunohistochemistry to quantitate PNUTS expression on a tissue microarray containing 17,747 clinical prostate cancer specimens. As compared to normal prostate epithelium, PNUTS expression was often higher in cancer. Among 12,235 interpretable tumors, PNUTS staining was negative in 21%, weak in 34%, moderate in 35%, and strong in 10% of cases. High PNUTS expression was associated with higher tumor stage, classical and quantitative Gleason grade, nodal stage, surgical margin, Ki67 labeling index, and early biochemical recurrence (*p* < 0.0001 each). PNUTS expression proved to be a moderate prognostic parameter with a maximal univariable Cox proportional hazard for PSA recurrence-free survival of 2.21 compared with 5.91 for Gleason grading. It was independent from established prognostic parameters in multivariable analysis. Comparison with molecular data available from earlier studies using the same TMA identified associations between high PNUTS expression and elevated androgen receptor expression (*p* < 0.0001), presence of *TMPRSS2:ER*G fusion (*p* < 0.0001), and 8 of 11 chromosomal deletions (3p13, 5q21, 8p21, 10q23, 12p13, 13q14, 16q24, and 17p13; *p* < 0.05 each). Particularly strong associations with PTEN and 12p13 deletions (*p* < 0.0001 each) may indicate a functional relationship, which has already been established for PNUTS and PTEN. PNUTS had no additional role on outcome in PTEN-deleted cancers. In conclusion, the results of our study identify high PNUTS protein levels as a predictor of poor prognosis possibly linked to increased levels of genomic instability. PNUTS measurement, either alone or in combination, might be of clinical utility in prostate cancers.

## 1. Introduction

In a recent overview from Bray et al., prostate cancer is the most common cancer in males in the majority of countries in the world [1]. The clinical course is highly variable, and active surveillance and watchful waiting are newer concepts in the therapy of prostate cancer [2]. Therefore, we need reliable criteria for the distinction between high-risk and low-risk patients. It is hoped that molecular prognostic markers will improve this selection.

Protein phosphatase 1 nuclear-targeting subunit (PNUTS) is a bifunctional gene encoding both the PNUTS protein and, by alternative splicing, a long noncoding RNA termed lncRNA-PNUTS [3]. The PNUTS protein is ubiquitously expressed and associates with protein phosphatase 1 (PP1) to control its activity [4]. PP1 is one of the major human protein phosphatases that dephosphorylate hundreds of kinases involved in a wide range of cellular actions [5]. Accordingly, PNUTS is implicated in all PP1-governed processes including RNA processing [6], DNA repair [7], chromatin decondensation [8], and maintenance of telomere stability [9]. PP1 is also involved in growth control through interaction with PTEN, RB1, MYC, and Aurora kinases [10–14]. The noncoding transcript lncRNA-PNUTS appears to function as a regulator of epithelial-mesenchymal transition, an essential process for tissue and organ formation, and also for tumor development and metastasis [15]. Several studies suggest that targeting PNUTS may have antitumor activity. For example, PNUTS knockdown increased apoptosis in breast, ovarian, and colon cancers cells [11] and potentiated the apoptotic effect of the cyclin-dependent kinase inhibitor roscovitine in breast and colon cancer cells [16]. Injection of the microRNA miR-34a, a natural downregulator of PNUTS, into glioblastoma xenograft tumors reduced telomere length [17] and targeting PNUTS with miR-383 induced cell cycle arrest in testicular embryonal carcinoma cells [18]. Most recently, it was shown that PNUTS is an essential partner of poly (ADP-ribose) polymerase 1 in DNA repair [19], making PNUTS a potential drug target in the therapy of DNA double-strand repair-deficient tumors.

One report using mRNA expression data from the Oncomine database [20] suggests that PNUTS may be upregulated in prostate cancers as compared to normal prostate tissues [10]. This observation prompted us to further investigate the role of this potentially important gene by analyzing a prostate cancer tissue microarray containing tumor samples from more than 17,000 individual patients.

## 2. Materials and Methods

### 2.1. Patients

The 17,747 patients had prostatectomy between 1992 and 2015 (Department of Urology and the Martini Clinic at the University Medical Center Hamburg-Eppendorf). Quantitative Gleason grading was performed as described [21]. Follow-up data were available for 14,667 patients with a median follow-up of 48 months. Prostate-specific antigen (PSA) recurrence was defined as the time point when postoperative PSA was at least 0.2 ng/ml and increasing at subsequent measurements. Patient characteristics are summarized in Table 1. The tissue microarray (TMA) manufacturing process was described earlier [22]. A single 0.6 mm core was taken for each patient. The TMA was annotated with data on Ki67 labeling index (Ki67LI) [23], ERG protein expression and ERG rearrangement analysis by fluorescence in situ hybridization (FISH) [24], and deletion status of 3p13 (FOXP1) [25], 5q21 (CHD1) [26], 6q15 (MAP3K7) [27], 8p21 [28], 10q23 (PTEN) [29], 12p13 [30], 12q24 [31], 13q14 [32], 16q24 [33], 17p13 [34], and 18q21 [35]. The usage of archived diagnostic leftover tissues for manufacturing of tissue microarrays and their analysis for research purposes as well as patient data analysis has been approved by local laws (HmbKHG, §12a) and by the local ethics committee (Ethics Commission Hamburg, WF-049/09). All work has been carried out in compliance with the Helsinki Declaration.

### 2.2. Immunohistochemistry

Freshly cut TMA sections were stained in a single experiment, deparaffinized, and exposed to heat-induced antigen retrieval (5 min, 121°C, pH 7.8 Tris-EDTA buffer). Primary antibody specific for PNUTS (rabbit polyclonal antibody, Novus Biologicals, Centennial, Colorado, USA, NB100-604; dilution 1 : 4,050) was applied (37°C, 60 min) and visualized with EnVision (Dako, Glostrup, Denmark). Anti-PNUTS showed punctuate staining inside the nuclei that was typically accompanied by weaker cytoplasmic costaining. Because the nuclear staining pattern is compatible with its function and cellular localization [36], only nuclear staining was scored as follows. The staining intensity (0, 1+, 2+, and 3+) as well as the fraction (percentage) of stained cells was recorded for each tissue spot. A final score was built from these two parameters as follows. Lack of any staining (intensity 0) was considered “negative,” 1+ staining in ≤70% of tumor cells or 2+ staining in ≤30% of tumor cells was considered “weak,” 2+ staining in >70% of tumor cells or 2+ staining in >30% but ≤70% of tumor cells or 3+ staining in ≤30% of tumor cells was considered “moderate,” and 2+ staining in >70% of tumor cells or 3+ staining in >30% of tumor cells was considered “strong.”

### 2.3. Statistics

JMP 12 software (SAS Institute Inc., NC, USA) was used for calculations. Contingency tables and the *χ*^2^ test were utilized to examine associations between molecular and histopathological tumor parameters. Kaplan-Meier survival curves were analyzed with the log-rank test to detect differences between groups. Cox proportional hazards regression analysis was performed to test for statistical independence between pathological, molecular, and clinical variables.

## 3. Results

### 3.1. Technical Issues

A total of 12,235 of 17,747 tumor samples (69%) were interpretable in our TMA analysis. Reasons for 31% noninformative cases included lack of tissue samples or absence of unequivocal cancer tissue in the TMA spot.

### 3.2. PNUTS Expression in Normal and Cancerous Prostate Tissues

**Figure 1:**
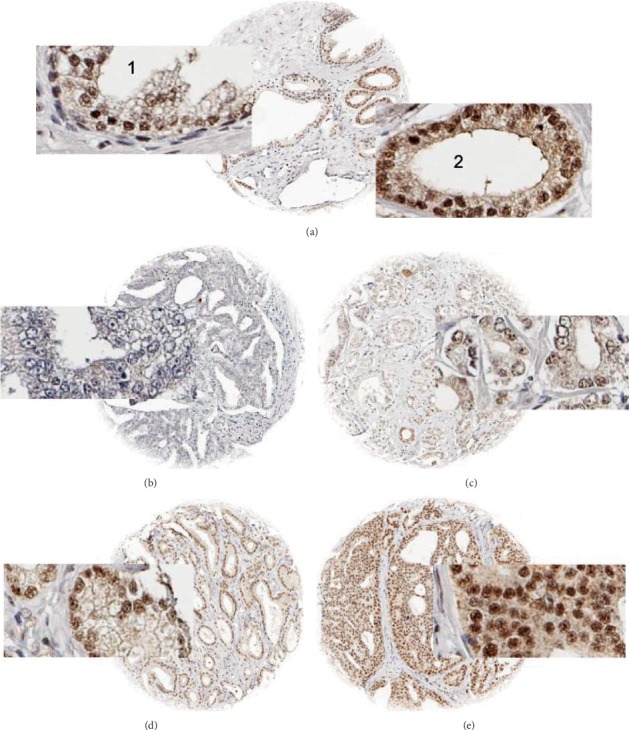
Examples of PNUTS staining: (a) comparison of PNUTS staining in (1) normal and (2) cancerous prostate glands of the same TMA spot. Cancer spots with (b) lack of staining and (c) weak, (d) moderate, and (e) strong staining.

**Figure 2:**
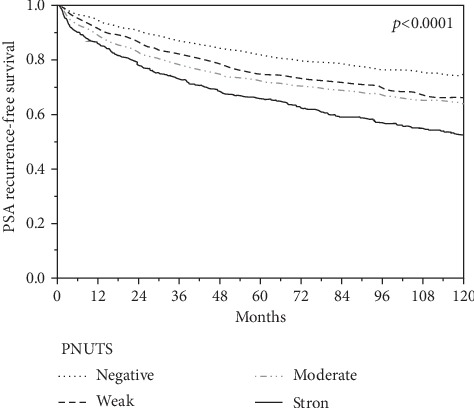
Association between PNUTS expression and biochemical recurrence after prostatectomy.

PNUTS staining was predominantly nuclear with a focus on nucleoli resulting in a “punctuated” staining pattern. In 20 cases of normal prostate gland, luminal cells stained weakly positive for PNUTS, while basal cells were negative (Figure 1(a1)). In prostate cancers, nuclear staining was seen in 9632 of our 12,235 (78.7%) interpretable tumors and was considered weak in 33.9%, moderate in 34.7%, and strong in 10.1% of cases (Figures 1(a2), 1(b), and 1(e)). Increased PNUTS staining showed significant associations with adverse tumor features (Table 2). Strong staining of PNUTS was associated with advanced tumor stage (*p* < 0.0001), high classical and quantitative Gleason grade (*p* < 0.0001 each), presence of lymph node metastasis (*p* < 0.0001), high preoperative PSA level (*p* = 0.0001), and positive surgical margin (*p* < 0.0001). PNUTS staining was also linked to early biochemical recurrence (Figure 2). Accordingly, subgroup analyses of cancers with identical Gleason score revealed a prognostic role of PNUTS expression in Gleason 3+4 (*p* < 0.0001) and Gleason 4+3 (*p* = 0.0002, Supplementary Figure S2a) as well as in particular quantitative Gleason grades (Supplementary Figure S2d, h).

### 3.3. PNUTS and *TMPRSS2:ERG* Fusion Status

**Figure 3:**
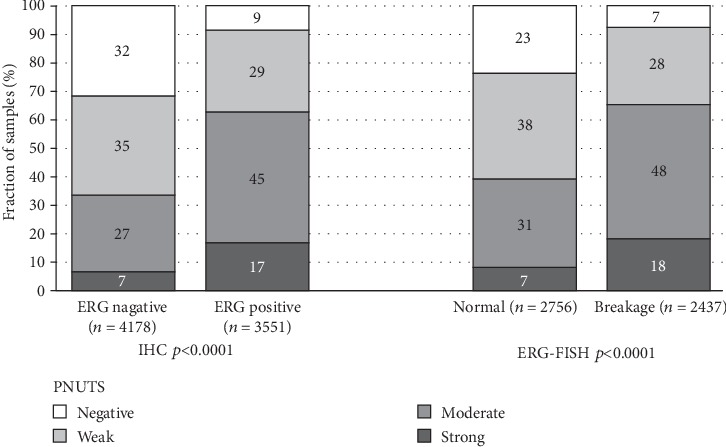
PNUTS staining and ERG status (IHC/FISH).

Data on both ERG FISH and IHC were available in 4,998 cancers with concordant results in 95% of cases. PNUTS staining was significantly more prevalent in cancers harboring *TMPRSS2:ERG* rearrangements than in cancer lacking ERG fusions (Figure 3). Because of these differences, associations of PNUTS with tumor phenotype and PSA recurrence were separately analyzed in ERG-negative and ERG-positive cancers. All these associations with tumor phenotype (Supplementary Tables S1 and S2) and clinical outcome (*p* < 0.0001 each, Supplementary Figure S1) hold true in both subgroups.

### 3.4. PNUTS and Chromosomal Deletions

For 10 of 11 analyzed deletions, PNUTS staining was stronger in deleted than in nondeleted cancers. This difference reached statistical significance for 8 of the 11 analyzed regions (Supplementary Figure S1). In ERG-negative cancers, a statistically significant difference was also seen for 8 of 11 analyzed deletions (Supplementary Figure S1b). In ERG-positive cancers, a statistically significant difference was found for 5 of 11 deletions (Supplementary Figure S1c). A particularly prominent PNUTS staining was seen in cancers with PTEN (10q23) and 12p deletions, which was highly significant in all, ERG-negative, and ERG-positive cancers (*p* < 0.0001 each).

### 3.5. PNUTS, Androgen Receptor, and Tumor Cell Proliferation (Ki67LI)

**Figure 4:**
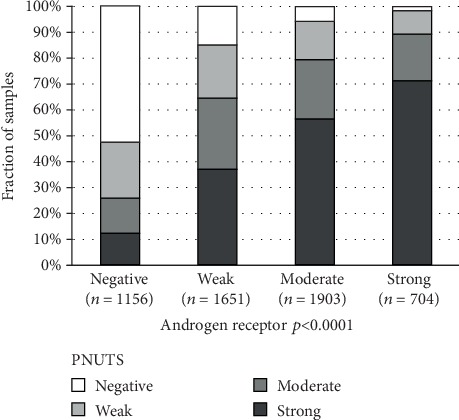
PNUTS staining and androgen receptor expression.

In 5,414 cancers with available AR expression data, the intensity of PNUTS staining was highly related to AR levels (Figure 4). This also applied for subsets of ERG-negative and ERG-positive cancers (data not shown). PNUTS staining was significantly linked to increased Ki67LI (Table 3). This held true in most subsets of cancers with identical Gleason score, including Gleason ≤3+3 (*p* < 0.0001), 3+4 (*p* < 0.0001), 3+4 with tertiary grade 5 (*p* = 0.0019), and 4+3 (*p* = 0.0009).

### 3.6. PSA Recurrence-Free Survival in Uni- and Multivariable Analyses

The univariable analysis showed a moderate Cox proportional hazard ratio of 2.21 for strong versus negative PNUTS expression compared with a hazard ratio of 5.91 for Gleason grading (Table 4). When the five variables of the univariable analysis were combined in the multivariable model, the hazard ratios were reduced but remained significant for each variable indicating that they are in part independent of each other. Strong PNUTS expression was predictive at a comparable level as PTEN deletion. The effect was observed in both ERG subsets (Table 4, Supplementary Table S3).

## 4. Discussion

The results of our study identify high PNUTS protein expression as a predictor of poor prognosis in prostate cancer. The peculiar “punctuated” nuclear staining pattern of PNUTS has already been described in an earlier study [36]. It fits well with the known interaction with PP1, which constitutes an important part of the nucleolar proteome. The PP1 subunit PP1 gamma and to a lesser extent PP1 beta are highly concentrated in the nucleoli of interphase cells [36]. Finding PNUTS expression at higher levels in cancer glands as compared to adjacent normal prostate gland suggests that the protein becomes upregulated during prostate cancer development and progression. Our findings are supported by findings from the Oncomine database, where PNUTS mRNA levels were higher in prostate cancers as compared to normal prostate tissues [10]. Additional expression data from the same database revealed PNUTS mRNA upregulation also in other tumor-normal pairs, including skin, kidney, and brain tissues. An immunohistochemical study also demonstrated PNUTS upregulation in esophageal carcinomas [10]. Altogether, these data suggest that PNUTS upregulation may accompany neoplastic transformation in many tumor types.

The association of high PNUTS staining with adverse tumor features, including advanced stage, high Gleason grade, nodal metastases, and early biochemical recurrence, argues for a role of PNUTS overexpression in prostate cancer progression. PNUTS is a protein that has not been extensively studied in cancer. A Medline search on 9^th^ March 2019 using the terms PNUTS and CANCER resulted in 19 citations. Data on a potential prognostic role of PNUTS activity in other cancer types is thus largely lacking. Kavela et al. reported that immunohistochemical PNUTS expression was stronger in 20 cases of triple-positive breast cancers that in 20 triple-negative breast cancers [10]. Overall, the known interaction with key genes in cancer including PTEN and PP1 regulating also RB1, MYC, MDM2, p53, and Aurora kinases is consistent with an important role of PNUTS in cancer [13, 37–40].

Further of note, PNUTS maps to chromosome 6p21.3 within the major histocompatibility complex (MHC) class I gene cluster and contains binding intervals for the MHC master regulator CIITA [41]. It cannot be excluded that PNUTS overexpression may also impact antigen presentation and lead to altered immune responses against cancer.

The highly annotated molecular database enabled us to compare PNUTS expression with other relevant molecular alterations. About 50% of prostate cancers contain gene fusions connecting the androgen-regulated *TMPRSS2* gene with the transcription factor *ERG* [31, 42]. These fusions result in a high-level androgen-dependent expression of ERG [43] eventually leading to an altered expression of more than 1,600 genes in prostate epithelial cells [44]. The significant upregulation of PNUTS in cancers having a *TMPRSS2:ER*G fusion demonstrates that PNUTS is either directly or indirectly impacted by ERG expression. Possible explanations for this relationship include the known interaction of PNUTS with PTEN [10]. ERG expression suppresses the transcription of PTEN [45]. The significant association between androgen receptor and PNUTS expression fits well with earlier reports demonstrating interactions between the PNUTS target PP1 and the androgen receptor.

Next to *TMPRSS2:ERG* fusions, genomic deletions at various chromosomal loci represent the most prevalent recurrent genetic alterations in prostate cancer where specific mutations of cancer genes are rare [46] [47] [31] [48]. Most chromosomal deletions either preferentially occur in ERG-negative (5q, 6q, 13q, and 18q) or in ERG-positive (3p, 8p *PTEN* (*10q23*), 12q, 16, and 17p) cancers. To exclude false statistical associations due to the link of both PNUTS expression and deletions to the ERG status, this analysis was also done for ERG-positive and ERG-negative cancers. That elevated PNUTS expression which was significantly associated with the majority of the analyzed deletions in ERG-negative cancers highlights that elevated PNUTS levels are either a cause or a consequence of genomic instability in prostate cancer cells. Given the pivotal interaction of PNUTS with PP1, an impact of PNUTS on the level of genomic stability appears plausible. Overexpression of NIPP1, another interaction partner of PP1, was earlier shown to limit a cell's capacity to repair DNA double-strand breaks [49]. Upregulation of PNUTS was most striking in cancers with PTEN and 12p13 deletions. In the case of PTEN, this may fit to the known functional interaction of both proteins. PNUTS binds directly to the PTEN protein and inactivates it through controlling its phosphatase activity. Knockdown of PNUTS in cell lines from prostate and other cancers resulted in apoptosis and growth arrest in a PTEN-dependent manner [10]. It could be hypothesized that high-level PNUTS expression may result in a particularly strong growth advantage in cancers with already reduced PTEN activity due to heterozygous deletion. However, this notion is not supported by the conspicuous lack of prognostic impact of PNUTS expression in 967 PTEN-deleted cancers. The tight relationship of elevated PNUTS expression and 12p13 deletions may pinpoint towards another relevant functional interaction. For example, ING4 maps to 12p13 and it is a tumor suppressor protein that contains a PHD-finger, which is a common motif in proteins involved in chromatin remodeling [50, 51]. Similarly, CHD4 is also in this target deleted region and it is also involved in chromatin remodeling [52, 53]. The data of this study identify high PNUTS expression as a strong and potentially clinically applicable prognostic marker in prostate cancer. The independent prognostic role of PNUTS expression was even retained if prognostic parameters were included, such as pT and pN stage, that are only available after prostatectomy and which are not known at the moment when therapeutic decisions are taken. That PNUTS expression which showed a prognostic impact in Gleason 3+4 (*p* < 0.0001) and Gleason 4+3 carcinomas (*p* = 0.0002) demonstrates that these morphologically defined subgroups include cancers with highly variable aggressiveness. We had earlier shown that subdividing Gleason 3+4 and 4+3 cancers according to their percentage of Gleason 4 pattern results in a much finer prediction of tumor progression [21]. The limited prognostic role of PNUTS expression in cancers with comparable quantitative Gleason grade underscores the potential prognostic power of an optimized morphological prostate cancer assessment. This also demonstrates how difficult it is for a prognostic molecular marker to compete with an optimized assessment of morphological prostate cancer features. It is of note, however, that there is not only a need for better predictors of PCA aggressiveness than the existing ones but also for more reproducible parameters. The Gleason grade, for example, suffers from an interobserver variability in the range of 40% [54, 55]. This issue is not fully solved by a quantitative Gleason grading approach. We thus expect that molecular analysis including multiple different features will in the future improve prognosis assessment.

## 5. Conclusions

In summary, the results of our study demonstrate that upregulation of PNUTS is tightly linked to aggressive tumor behavior and poor prognosis in prostate cancer. Measuring PNUTS expression either alone or in combination with other prognostic markers might have clinical utility in prostate cancer.

## Figures and Tables

**Table 1:** Pathological and clinical data of the arrayed prostate cancers.

	No. of patients (%)	
	Study cohort on TMA^∗^	Biochemical relapse
*Follow-up*	14,464	3,612 (25%)
Mean/median (month)	56/48	—
*Age* (*y*)		
≤50	433	66 (15%)
51-59	4,341	839 (19%)
60-69	9,977	2,073 (21%)
≥70	2,936	634 (22%)
*Pretreatment PSA* (*ng/ml*)		
<4	2,225	313 (14%)
4-10	10,520	1,696 (16%)
10-20	3,662	1,043 (29%)
>20	1,231	545 (44%)
*pT stage* (*AJCC 2002*)		
pT2	11,518	1,212 (11%)
pT3a	3,842	1,121 (29%)
pT3b	2,233	1,213 (54%)
pT4	85	63 (74%)
*Gleason grade*		
≤3+3	3,570	264 (7%)
3+4	9,336	1,436 (15%)
3+4 tertiary 5	1,697	165 (10%)
4+3	2,903	683 (24%)
4+3 tertiary 5	1,187	487 (41%)
≥4+4	999	531 (53%)
*pN stage*		
pN0	10,636	2,243 (21%)
pN+	1,255	700 (56%)
*Surgical margin*		
Negative	14,297	2,307 (16%)
Positive	3,388	1,304 (39%)

^∗^Numbers do not always add up to 17,747 in different categories because of cases with missing data. AJCC: American Joint Committee on Cancer.

**Table 2:** PNUTS expression and cancer phenotype.

Parameter	*N*	PNUTS expression (%)				*p*
		Negative	Weak	Moderate	Strong	
*All cancers*	12,235	21	34	35	10	
*Tumor stage*						<0.0001
pT2	7,895	25	36	32	8	
pT3a	2,707	17	30	38	14	
pT3b-pT4	1,590	12	31	42	14	
*Gleason grade*						<0.0001
≤3+3	2,379	34	31	28	7	
3+4	6,559	20	36	34	10	
3+4 tertiary 5	549	15	37	40	8	
4+3	1,168	14	31	40	15	
4+3 tertiary 5	867	10	34	44	13	
≥4+4	619	15	29	42	14	
*Quantitative Gleason grade*						<0.0001
≤3+3	2,379	34	31	28	7	
3+4 ≤5%	1,682	24	39	30	7	
3+4 6-10%	1,639	22	36	33	9	
3+4 11-20%	1,466	18	35	35	12	
3+4 21-30%	737	16	33	39	13	
3+4 31-49%	570	17	36	37	10	
3+4 tertiary 5	549	15	37	40	8	
4+3 50-60%	501	14	34	39	14	
4+3 tertiary 5	867	10	34	44	13	
4+3 61-100%	521	15	29	40	16	
≥4+4	542	15	31	41	13	
*Lymph node metastasis*						<0.0001
N0	7,347	18	34	37	11	
N+	856	10	31	44	15	
*Preoperative PSA level* (*ng/ml*)						0.0001
<4	1,469	20	36	33	11	
4-10	7,293	21	35	35	9	
10-20	2,546	22	33	35	10	
>20	854	22	28	37	14	
*Surgical margin*						<0.0001
Negative	9,771	22	35	34	9	
Positive	2,423	18	30	39	13	

**Table 3:** PNUTS staining correlates with Ki67 labeling index.

Gleason	PNUTS	*N*	Ki67LI (mean ± SEM)	*p*
All	Negative	1,181	1.74 ± 0.07	<0.0001
	Weak	1,695	2.71 ± 0.06	
	Moderate	1,831	3.02 ± 0.06	
	Strong	598	3.82 ± 0.10	
≤3+3	Negative	386	1.46 ± 0.10	<0.0001
	Weak	349	2.42 ± 0.11	
	Moderate	345	2.43 ± 0.11	
	Strong	92	3.41 ± 0.21	
3+4	Negative	622	1.69 ± 0.09	<0.0001
	Weak	968	2.54 ± 0.07	
	Moderate	1,065	2.86 ± 0.07	
	Strong	336	3.55 ± 0.12	
3+4 tertiary 5	Negative	32	1.75 ± 0.42	0.0019
	Weak	82	3.35 ± 0.26	
	Moderate	70	3.71 ± 0.29	
	Strong	14	2.93 ± 0.64	
4+3	Negative	78	2.29 ± 0.36	0.0009
	Weak	150	3.41 ± 0.26	
	Moderate	183	3.36 ± 0.24	
	Strong	80	4.38 ± 0.36	
4+3 tertiary 5	Negative	30	3.37 ± 0.70	0.1913
	Weak	90	3.34 ± 0.40	
	Moderate	105	4.35 ± 0.37	
	Strong	42	4.45 ± 0.59	
≥4+4	Negative	33	3.24 ± 0.80	0.0528
	Weak	56	3.66 ± 0.62	
	Moderate	61	4.97 ± 0.59	
	Strong	33	5.88 ± 0.80	

**Table 4:** Cox proportional hazards for PSA recurrence-free survival after prostatectomy of established preoperative prognostic parameter and PNUTS expression.

Variable		*N*	Univariable analysis	Multivariable analysis (*N* = 4,392)
Gleason grade biopsy	≥4+4 vs. ≤3+3	12,172	5.91 (5.33-6.55)^∗∗∗^	3.75 (3.13-4.49)^∗∗∗^
cT stage	T2c vs. T1c	14,404	2.15 (1.72-2.65)^∗∗∗^	2.00 (1.46-2.74)^∗∗∗^
Preoperative PSA level	≥20 vs. <4	14,611	5.06 (4.41-5.81)^∗∗∗^	3.69 (2.80-4.86)^∗∗∗^
PTEN	Deletion vs. normal	6,236	2.10 (1.89-2.33)^∗∗∗^	1.44 (1.26-1.65)^∗∗∗^
PNUTS expression	Strong vs. negative	10,029	2.21 (1.93-2.53)^∗∗∗^	1.61 (1.30-2.01)^∗∗∗^
ERG-negative subset	Strong vs. negative	4,342	2.70 (2.17-3.36)^∗∗∗^	—
ERG-positive subset	Strong vs. negative	3,811	2.16 (1.67-2.83)^∗∗∗^	—

Confidence interval (95%) in brackets; asterisks indicate significance level: ^∗^*p* ≤ 0.05, ^∗∗^*p* ≤ 0.001, and ^∗∗∗^*p* ≤ 0.0001.

## Data Availability

Answer: No. Comment: The data used to support the findings of this study are available from the corresponding author upon request.

## Supplementary Materials

Supplementary Materials Supplementary Table S1: PNUTS staining and prostate cancer phenotype in ERG-negative cancers. Supplementary Table S2: PNUTS staining and prostate cancer phenotype in ERG-positive cancers. Supplementary Table S3: multivariate Cox regression analysis including established prognostic parameters and the PNUTS expression in all prostate cancers, the ERG-*negative* and ERG-*positive* subsets. Supplementary Fig. S1: association between PNUTS immunostaining and common chromosomal deletions in (a) all cancer, (b) ERG-negative cancers, and (c) ERG-positive cancers. Supplementary Fig. S2: prognostic impact of PNUTS defined by the Gleason score. (a) Impact of PNUTS expression as compared to the classical Gleason score categories. Impact of expression as compared to the quantitative Gleason score categories defined by subsets of cancers with (b) ≤5% Gleason 4 patterns, (c) 6-10% Gleason 4 patterns, (d) 11-20% Gleason 4 patterns, (e) 21-30% Gleason 4 patterns, (f) 31-49% Gleason 4 patterns, (g) 50-60% Gleason 4 patterns, and (h) ≥61% Gleason 4 patterns.
