# Supplementary material for: Upregulation of Phosphatase 1 Nuclear-Targeting Subunit (PNUTS) Is an Independent Predictor of Poor Prognosis in Prostate Cancer
Source: Dis Markers. 2020 Apr 25;2020:7050146. doi: 10.1155/2020/7050146 (PMC7196962; doi:10.1155/2020/7050146)
Supplement: Supplementary Materials — Supplementary Table S1: PNUTS staining and prostate cancer phenotype in ERG-negative cancers. Supplementary Table S2: PNUTS staining and prostate cancer phenotype in ERG-positive cancers. Supplementary Table S3: multivariate Cox regression analysis including established prognostic parameters and the PNUTS expression in all prostate cancers, the ERG-negative and ERG-positive subsets. Supplementary Fig. S1: association between PNUTS immunostaining and common chromosomal deletions in (a) all cancer, (b) ERG-negative cancers, and (c) ERG-positive cancers. Supplementary Fig. S2: prognostic impact of PNUTS defined by the Gleason score. (a) Impact of PNUTS expression as compared to the classical Gleason score categories. Impact of expression as compared to the quantitative Gleason score categories defined by subsets of cancers with (b) ≤5% Gleason 4 patterns, (c) 6-10% Gleason 4 patterns, (d) 11-20% Gleason 4 patterns, (e) 21-30% Gleason 4 patterns, (f) 31-49% Gleason 4 patterns, (g) 50-60% Gleason 4 patterns, and (h) ≥61% Gleason 4 patterns. [file 7050146.f1.docx]

**Supplement**

**Table S1** PNUTS staining and prostate cancer phenotype in ERG negative cancers.

|  |  | **PNUTS expression (%)** | | | |  |
| --- | --- | --- | --- | --- | --- | --- |
| **Parameter** | **N** | **Negative** | **Weak** | **Moderate** | **Strong** | **P** |
| **All cancers** | 4,178 | 32 | 35 | 27 | 7 |  |
|  |  |  |  |  |  |  |
| **Tumor stage** |  |  |  |  |  | <0,0001 |
| pT2 | 2,850 | 35 | 36 | 25 | 5 |  |
| pT3a | 826 | 28 | 32 | 29 | 11 |  |
| pT3b-pT4 | 492 | 20 | 33 | 35 | 13 |  |
|  |  |  |  |  |  |  |
| **Gleason grade** |  |  |  |  |  | <0.0001 |
| ≤3+3 | 852 | 45 | 27 | 22 | 5 |  |
| 3+4 | 2,263 | 32 | 37 | 25 | 6 |  |
| 3+4 Tertiary 5 | 175 | 21 | 43 | 31 | 5 |  |
| 4+3 | 418 | 22 | 34 | 33 | 11 |  |
| 4+3 Tertiary 5 | 256 | 16 | 39 | 36 | 9 |  |
| ≥4+4 | 212 | 22 | 31 | 33 | 14 |  |
|  |  |  |  |  |  |  |
| **Quantitative Gleason grade** |  |  |  |  |  |  |
| ≤3+3 | 852 | 45 | 27 | 22 | 5 | <0.0001 |
| 3+4 ≤5% | 603 | 38 | 36 | 22 | 4 |  |
| 3+4 6-10% | 591 | 35 | 38 | 23 | 4 |  |
| 3+4 11-20% | 519 | 30 | 37 | 25 | 8 |  |
| 3+4 21-30% | 258 | 24 | 38 | 29 | 9 |  |
| 3+4 31-49% | 211 | 24 | 36 | 32 | 9 |  |
| 3+4 Tert.5 | 175 | 21 | 43 | 31 | 5 |  |
| 4+3 50-60% | 186 | 22 | 38 | 33 | 7 |  |
| 4+3 Tert.5 | 256 | 16 | 39 | 36 | 9 |  |
| 4+3 61-100% | 200 | 22 | 34 | 31 | 13 |  |
| ≥4+4 | 195 | 21 | 33 | 33 | 13 |  |
|  |  |  |  |  |  |  |
| **Lymph node metastasis** |  |  |  |  |  | 0.0113 |
| N0 | 2,434 | 28 | 35 | 29 | 8 |  |
| N+ | 214 | 19 | 36 | 33 | 13 |  |
|  |  |  |  |  |  |  |
| **Preoperative PSA level (ng/ml)** |  |  |  |  |  | 0.0028 |
| <4 | 437 | 29 | 34 | 27 | 10 |  |
| 4-10 | 2,502 | 32 | 36 | 26 | 6 |  |
| 10-20 | 912 | 32 | 33 | 27 | 8 |  |
| >20 | 306 | 33 | 28 | 27 | 11 |  |
|  |  |  |  |  |  |  |
| **Surgical margin** |  |  |  |  |  | 0.0006 |
| Negative | 3,340 | 32 | 35 | 26 | 6 |  |
| Positive | 828 | 28 | 32 | 30 | 10 |  |

**Table S2** PNUTS staining and prostate cancer phenotype in ERG positive cancers.

|  |  | **PNUTS expression (%)** | | | |  |
| --- | --- | --- | --- | --- | --- | --- |
| **Parameter** | **N** | **Negative** | **Weak** | **Moderate** | **Strong** | **P** |
| **All cancers** | 3,551 | 9 | 29 | 45 | 17 |  |
|  |  |  |  |  |  |  |
| **Tumor stage** |  |  |  |  |  | <0.0001 |
| pT2 | 2,152 | 10 | 31 | 45 | 14 |  |
| pT3a | 942 | 6 | 27 | 46 | 20 |  |
| pT3b-pT4 | 445 | 7 | 26 | 46 | 22 |  |
|  |  |  |  |  |  |  |
| **Gleason grade** |  |  |  |  |  | <0.0001 |
| ≤3+3 | 735 | 16 | 30 | 41 | 13 |  |
| 3+4 | 2,058 | 8 | 30 | 46 | 17 |  |
| 3+4 Tertiary 5 | 106 | 4 | 38 | 46 | 12 |  |
| 4+3 | 351 | 4 | 25 | 46 | 25 |  |
| 4+3 Tertiary 5 | 190 | 5 | 26 | 51 | 19 |  |
| ≥4+4 | 109 | 8 | 25 | 46 | 21 |  |
|  |  |  |  |  |  |  |
| **Quantitative Gleason grade** |  |  |  |  |  | <0.0001 |
| ≤3+3 | 735 | 16 | 30 | 41 | 13 |  |
| 3+4 ≤5% | 520 | 10 | 34 | 44 | 12 |  |
| 3+4 6-10% | 547 | 9 | 29 | 46 | 16 |  |
| 3+4 11-20% | 457 | 5 | 31 | 43 | 21 |  |
| 3+4 21-30% | 257 | 5 | 23 | 50 | 21 |  |
| 3+4 31-49% | 170 | 8 | 33 | 46 | 13 |  |
| 3+4 Tert.5 | 106 | 4 | 38 | 46 | 12 |  |
| 4+3 50-60% | 160 | 3 | 34 | 40 | 23 |  |
| 4+3 Tert.5 | 190 | 5 | 26 | 51 | 19 |  |
| 4+3 61-100% | 157 | 4 | 18 | 52 | 25 |  |
| ≥4+4 | 91 | 8 | 27 | 45 | 20 |  |
|  |  |  |  |  |  |  |
| **Lymph node metastasis** |  |  |  |  |  | 0.5154 |
| N0 | 2,045 | 6 | 27 | 48 | 19 |  |
| N+ | 206 | 5 | 25 | 47 | 23 |  |
|  |  |  |  |  |  |  |
| **Preoperative PSA level (ng/ml)** |  |  |  |  |  | 0.0008 |
| <4 | 477 | 8 | 33 | 41 | 18 |  |
| 4-10 | 2,197 | 9 | 30 | 46 | 15 |  |
| 10-20 | 633 | 9 | 26 | 45 | 19 |  |
| >20 | 219 | 11 | 20 | 45 | 24 |  |
|  |  |  |  |  |  |  |
| **Surgical margin** |  |  |  |  |  | <0.0001 |
| Negative | 2,812 | 9 | 31 | 44 | 15 |  |
| Positive | 726 | 7 | 22 | 48 | 23 |  |

**Table S3** Multivariate Cox regression analysis including established prognostic parameters and the PNUTS expression in all prostate cancers, the ERG *negative* and ERG *positive* subset.

**Fig. S1** Association between PNUTS immunostaining and common chromosomal deletions in a) all cancer, b) in ERG negative cancers and c) in ERG positive cancers

**Fig. S2** Prognostic impact of PNUTS defined by the Gleason score: a) Impact of PNUTS expression as compared to the classical Gleason score categories. b-h) Impact of expression as compared to the quantitative Gleason score categories defined by subsets of cancers with b) ≤5% Gleason 4 patterns, c) 6-10% Gleason 4 patterns, d) 11-20% Gleason 4 patterns, e) 21-30% Gleason 4 patterns, f) 31-49 % Gleason 4 patterns, g) 50-60% Gleason 4 patterns, h) ≥61% Gleason 4 patterns.
